# The threat of thinking in threats: reframing global health during and after COVID-19

**DOI:** 10.1007/s42597-020-00049-7

**Published:** 2020-11-09

**Authors:** Elena Sondermann, Cornelia Ulbert

**Affiliations:** Institut für Entwicklung und Frieden (INEF), Universität Duisburg-Essen, Lotharstr. 53, Duisburg, 47057 Germany

**Keywords:** Global health security, Global health solidarity, Vulnerabilities, Logic of exceptionalism, Infectious diseases, Globale Gesundheitssicherheit, Globale Gesundheitssolidarität, Vulnerabilitäten, Logik des Ausnahmezustands, Übertragbare Krankheiten

## Abstract

Narratives and metaphors shape how actors perceive the world around them and how policymakers frame the range of policy choices they think of as feasible. The metaphor of war and the narrative of how to tackle the unprecedented threat of COVID-19 are effective mechanisms to convey urgency. However, they also bear serious implications: Thinking in terms of health threats works with a logic of exceptionalism, which supports images of “us” vs. an “enemy” thereby shortening complex lines of causality and responsibility and privileging national answers. It fails to provide for a normative framework for drafting long-term systemic approaches. In this contribution, we critically engage with existing narratives of global health security and show how the logic of exceptionalism is limiting the current responses to the pandemic. We conceptualize an alternative narrative that is based on the logic of solidarity and argue that within this alternative framing a more sustainable and ultimately more just way of coping with infectious diseases will be possible.

## Abstract (de)

Narrative und Metaphern prägen, wie Akteure die Welt wahrnehmen und welche Handlungsspielräume für Politiker*innen möglich erscheinen. Das Bild des Krieges und das Narrativ der nie dagewesenen Bedrohung durch COVID-19 vermitteln wirkungsvoll die Dringlichkeit der Situation. Damit sind aber auch schwerwiegende Folgen verbunden: Ein Denken in Kategorien von „Gesundheitsgefahren“ geht mit einer Logik des Ausnahmezustands einher, die Vorstellungen von „uns“ gegen einen „Feind“ befördert. Dadurch werden komplexe Kausal- und Verantwortungsketten unzulässig verkürzt und eine rein nationale Vorgehensweise privilegiert. Das schafft jedoch keine normative Grundlage für langfristige, systemische Herangehensweisen. Im vorliegenden Beitrag setzen wir uns kritisch mit Narrativen globaler Gesundheitssicherheit auseinander und diskutieren, wie die „Handlungslogik des Ausnahmezustands“ die derzeitigen Reaktionen auf COVID-19 prägt und begrenzt. Wir skizzieren ein alternatives Narrativ, das sich auf die „Logik von Solidarität“ stützt, und zeigen, dass damit politische Vorgehensweisen einher gehen werden, die einen nachhaltigeren und letztendlich gerechteren Umgang mit übertragbaren Krankheiten ermöglichen.

## Introduction

Political speeches, media reporting and public debates are replete with notions of “the battle against Corona”, a “(global) war” (e.g. Harari 2020) and the fight against the “hidden enemy” (White House 2020a) or “invisible killer” (Prime Minister’s Office 2020). The narratives of war and the way to *fight* the COVID-19-enemy conveys urgency and prioritizes emergency measures. Moreover, they imply the hope of a “win”, thus an endpoint, as well as the idea that control and action are possible if all rally and pull together. All these notions seem obvious, smart and even necessary in the face of the worst infectious disease the world has experienced since the Spanish Flu of 1918, which infected about one-third (around 500 million people) (WHO 2018) of the world’s population at that time throughout three waves that lasted until 1920, taking up to 50 millions of lives (Taubenberger et al. 2019, p. 1) and rendering many more seriously ill or exposed to serious health risks.

Yet, these images and stories have other effects, too. Thinking in a *war on disease* frame creates images of “us” vs. “it” as a unified “we” against an *outside*, suddenly emerging *threat*. It silences differences within societies and nations, shortens lines of causality and entanglement and blurs responsibility. The sense of exceptionalism that is conveyed with this narrative easily links with a framing of health as an issue of security. Consequently, reactions are based on an emergency mode, thus preventing to act with a view to long-term systemic approaches.

We base our following discussion on the constructivist premise that the political implications of events are not simply there to be “discovered” and “told” but lie in the interpretations of (political) actors (e.g. Krebs 2015, p. 810). Framing is “understood as the presentation of an issue in such a way as to tie it into a broader set of ideas about the world” (McInnes and Roemer-Mahler 2017, p. 1319). Thereby, frames give meaning to events and processes by highlighting some aspects and silencing others. At the same time, frames usually comprise dominant logics of actions that pave the way for how a perceived problem will be tackled.

Framing often resorts to narratives which can be analyzed along three dimensions (Spencer 2016, p. 22–35). *Setting* refers to the broader context and background of the story providing associations and possible connotations for the audience. *Characterization* introduces protagonists and their quality and roles (i.e. hero or villain) in the story. Lastly, *emplotment* weaves them together by tying them to a causal origin, logical sequence of acts and probable consequences for action. For our discussion, we will use these categories for the analysis and differentiation of narratives. Firstly, we lay out the *setting*, i.e. the context for the framing of COVID-19 as “security threat” referring to previous framings of health as security, i.e. health securitization (Sect. 2). We then introduce the “COVID-19 as security threat” frame as “villain” (*characterization*) and show how this leads to a narrowing of the debate and political answers (*emplotment*) (Sect. 3). In a final move, we present an alternative narrative that is not based on the logic of exceptionalism but on the logic of solidarity (Sect. 4), and argue that within this alternative framing a more sustainable and ultimately more just way of coping with infectious diseases will be possible. Finally, we summarize the differing narratives of global health and the implications of their logics of action (Sect. 5).

## Framing health as security: competing narratives and implications

Health, illness and disease have always been object to framing processes: As health historians have long been drawing attention to, “disease” is not only a physical experience of illness but it is always also a social phenomenon as it represents the result of cultural sense-making and framing (e.g. Rosenberg 1989). Throughout the last three decades health issues have been increasingly framed as security concerns (e.g. Brown and Harman 2011). This was part of an overall development to move the concept of security beyond traditional inter-state conflicts and the notion of threat beyond military threats to include new security challenges. Driven by the emergence of the novel HIV/AIDS disease and followed by SARS, MERS, Ebola and Zika the “threat of diseases” was pushed up high on the international agenda (Rushton 2011). Infectious diseases have been debated in the UN General Assembly on numerous occasions and HIV/AIDS and Ebola have been addressed by UN Security Council resolutions as threats to national stability as well as national and international security (McInnes and Rushton 2010). Attention given to infectious diseases was intricately linked to and supported by the increased perception of health threats due to globalization and the associated, ever increasing speed of travel, transport and, relatedly, spread of pathogens. This framing of health issues as threats evoked connotations of something “out of the ordinary”, an exceptional danger to lives or countries’ stability, economies and trade. In light of this setting or broader context, the next sections discuss two variants of health narratives which entail different interpretations of the setting and, accordingly, competing characterizations and emplotments.

### Competing narratives of health security

Two competing narratives of health security gained ground at the turn of the century. Already in their characterization of the main protagonists they differ: a state-centric perspective of health security equating health issues to other threats to nation states and an alternative narrative of health security referring to individuals as the object of security (i.e. security for whom?). Health security from this latter perspective formed an integral part of the human security concept introduced by the United Nations Development Programme (UNDP) in the mid-1990s (UNDP 1994). While both narratives, what Sara Davies (2010) has labelled a “statist” (1) and a “globalist” perspective (2), refer to health security, they tell very different stories about whom to protect (states (1) vs. individuals (2)) and what to protect them from (mainly infectious diseases (1) vs. range of communicable and non-communicable diseases: “illness, disability and avoidable death” (Commission on Human Security 2003, p. 96) (2)). The second understanding characterizes, the “threat to human security” not as stemming from the “outside”. Rather, it views health threats as a direct result from structures of poverty and inequality i.e. in the form of health system access. External threats might be neither preventable nor controllable, hence changeable. Yet, internal structures certainly are. Thus, the role and responsibility of the state is portrayed very differently. In the first variant of the health security narrative states are assigned the role of insurers or rescuers of health security, while in the second variant they become part of the problem (“the villain”), if they wait until the threats to human health security materialize. It also entails an active and larger role for international organizations (IOs) and non-state actors. Regarding health policies, the globalist narrative leads to a stronger focus on strengthening health systems and ensuring equal access. Here, the idea of security has been stretched far, translating it to “freedom from want” and “freedom from fear”. Nonetheless, both narratives still characterize states as main protagonists, albeit with substantially different roles.

### Implications and limitations of the logic of exceptionalism

**Fig. 1:**
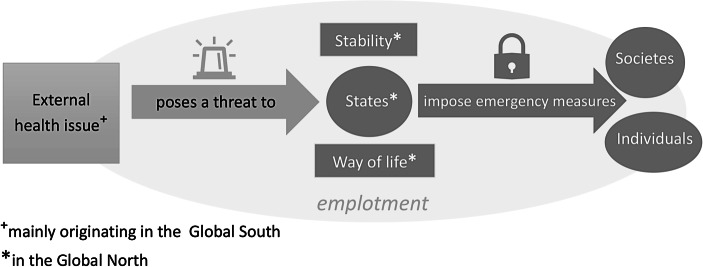
The logic of exceptionalism in the (narrow) health security narrative. (Source: Authors)

Through the linkage of health to “security against threats” the narrative of health security operates with a logic of exceptionalism: (external, also distant) health issues (i.e. infectious diseases) are perceived as positing severe or extraordinary danger to the physical well-being of individuals or entire societies, a threat to the normal (economic, cultural, financial) way of life in a country. This is framed as cause for political reactions in the form of emergency measures. Due to their linkage of health to security they always remain defensive. Their main frame is “ensuring health against a threat”. To date the narrow conceptualization of securing against infectious diseases dominates the mainstream policy agenda (see Fig. 1).

Yet, the core controversy lies in the questions of protecting whom, from what and at what cost. We address four implications and limitations of the narrative, which challenge its simplistic characterizations and emplotments and prepare our discussions for the subsequent parts.

First, we contend that exceptionalism itself means *substantially different* things to different actors. To countries of the Global North, the threats were exceptional in the sense of “new” as they realized their own, increased vulnerability and feared that for the first time in decades, “colonial problems” might reach across *their* borders and endanger *their* societies. However, in contrast to some countries of the Global South, to which infectious diseases are well known and even systemic, the health emergencies only very rarely threatened the security of countries of the Global North and their societies directly in the form of taking people’s lives.

Second, the logic of exceptionalism is *performative* and leads to a range of reactive policy options, which revolve around the object of fear, i.e. the threat. They are neither concerned with addressing root causes of the emergence of diseases nor with structural factors promoting vulnerability to exposure or the ability to cope.

Third, by centering on the object to secure and the external threat, neither the relations *between* objects of security nor their positional differences *in relation to* the threat are integral to the concept. This is true even for a more collective understanding of global health security as promoted by the World Health Organization (WHO) since the early 2000s (WHO 2007). Given the significant differences of countries regarding their exposure to, history of and coping capacities for infectious diseases “global health security” cannot mean the same for all countries. It also takes an unequal toll on countries. Emergency response measures, such as lock-down, closing the border and suspension of free flow of goods and services are mainly imposed on countries experiencing the disease, i.e.—before COVID-19—countries of the Global South. Hence, we argue that the manifold roles and responsibilities for realizing health security remain hidden as well as the diverging extent of the threat or costs for prevention and containment.

Lastly, exception per se means a *deviation from the norm*. Even though prevention and surveillance mechanisms can be thought of as longer-term governance practices, the anchoring concept of the health security governance regime is not. Threat in its nature is never a “normal” condition but works with individual and collective shorter-term notions of danger and fear. Thus, the global health security narrative does not entail a logic of action, which rests on normative, longer-term perspectives.

The narrative of exceptional global threats to national and international stability has certainly fostered attention and resources (Wenham 2019, p. 1106), at least for some time. Yet, the “‘global’ rhetoric” (Rushton 2011, p. 780) has not replaced traditional ideas of national and international health security. Instead, we contend that while the “global” in global health signaled a new awareness of shared “threats” and “risks” (Kirk 2020; McInnes and Roemer-Mahler 2017), the nation state, i.e. “our national health” has remained the reference object. This narrative of securing against infectious diseases is inherent to mainstream framing of health security and has provided the context and repertoire for emplotment regarding the Coronavirus pandemic.

## The limits of exception: a critique of COVID-19 security narratives

Since the WHO declared COVID-19 a pandemic on March 11, 2020, the subsequent narratives have been replete with notions of “existential threat”, “war” and “invisible enemy”. They are deeply entrenched in narratives of health security (setting). The protagonists are clear: “We” against an identifiable, outside threat, an “object of fear”, i.e. the Coronavirus. Yet, the more specific characterization of “we”, the reference object, was conceived of differently. While IO leaders, namely United Nations (UN) Secretary-General, António Guterres, referred to a “*common* enemy” and an “enemy of *humanity*” or to the “citizens of the world” (UN Secretary-General 2020; emphasis added), many political leaders were quick to jump to a narrative of national security and national emergency (Benziman 2020). The emplotment, thus the framing of the consequences for action, also diverged. Leaders of IOs promoted “international cooperation between governments and global coordination of policy responses” and cautioned that “all countries must strike a fine balance between protecting health, minimizing economic and social disruption, and respecting human rights” (WHO Director-General 2020). At the same time, U.S. President Donald J. Trump saw the United States “at war” and himself as a “war-time president” (White House 2020b). By describing COVID-19 as the “Chinese Virus” or “Wuhan Virus”, he framed the health emergency as a traditional security issue and another country as a source of that threat.

While these framings arguably constitute extremes of a continuum and can only be partly explained by the different roles and audiences of the speakers, it is striking how they all work with the image of an *existential* threat (albeit to a different “who”). This triggered the logic of exceptionalism inherent in the “health as security” narrative. The high transmission rate of the Coronavirus and the severity of the COVID-19 disease seem reason enough to resort to the logic of exceptionalism. However, the response to a disease always not only reflects the characteristics of the pathogen but also depends on the narration of the disease.

COVID-19 is a global pandemic and this universal experience and perceived “sameness” marks the crucial difference from any epidemic of the post-war history (Sondermann 2020). Yet, one could also challenge this notion by emphasizing that there have been pandemics in the recent past, however, not in industrialized countries. For some, the pandemic is thus more exceptional than for others. Not surprisingly, experiences, best practices and advice of countries of the Global South which had suffered from Ebola, Cholera, HIV/AIDS or Zika were largely being ignored (Harman 2020).

The narrative of the “exceptional threat to our live” has legitimized unprecedented political measures of physical distancing and lock-down all around the globe. They have disrupted not only the everyday lives of people worldwide but put to a halt all political, cultural and economic processes, both nationally and internationally. Following Benziman (2020) we argue that thinking in threats entails known categories of “us” vs “it”, an identifiable, *outside* threat, an “*object* of fear”. It presents the pandemic as something unforeseeable and “not our fault” (Harman 2020) and leads to a focus on reactive emergency measures, renewed agency and transmits a sense of control (emplotment). This shift to the executive has then led to a perpetuated cycle of securitizing health. This implies a narrow framing, which overlooks the health issues that actually account for the health matter at hand, namely health systems and access to health.

While Corona is a *global* health crisis it has been met in national understandings and corresponding national responses. These were mostly exclusively focused on protecting own citizens or framed as enhancing national interests. Moreover, the costs of the pandemic and containment measures differ significantly and will continue to do so. Emergency measures bear the risk of diminished civil rights accompanied by an increase in conflicts and humanitarian crises. Furthermore, other severe health crises loom as aid resources are redirected and vaccinations drop. All in all, inequalities between and within societies have manifested.

The outbreak of a Coronavirus pandemic was neither unexpected nor unanticipated. Instead, scenarios had been developed to envision and prepare for exactly such a pandemic. Yet, years of political and academic attention to the health-security nexus had neither prevented existing institutions for surveillance and control from being cut or diminished in recent years, nor did they appear to have succeeded in equipping the world’s countries for dealing with a global pandemic.

Therefore, we propose a reframing of health, which grapples with the complexity of global health as an intersectional issue and right of individuals. As we have seen earlier, similar ideas have been championed for decades. We are convinced that the current moment in time has opened a unique window of opportunity to overcome a narrow framing of health security. The current pandemic has shed light on the weaknesses of the idea of exceptionalism and the logic of action it entails. Thus, a change of setting, protagonists and overall storyline is needed to open up new courses of action.

## Reframing health: from security to solidarity

**Fig. 2:**
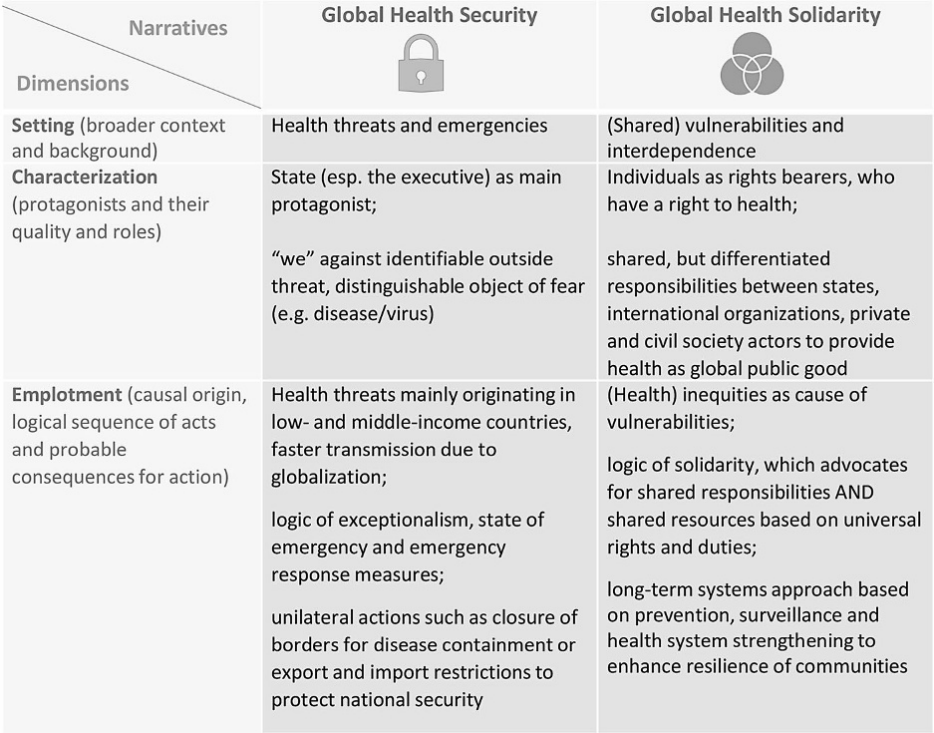
From Global Health Security to Global Health Solidarity. (Source: Authors)

It is highly likely that in the future humankind will have to cope with more incidents of infectious diseases transgressing borders and spreading globally (Bloom et al. 2017). This is not only due to the increasing mobility of goods and people, but also to our way of production and consumption that leads to environmental changes like climate change, land degradation or biodiversity loss. Although experts have been aware of these threats, which have been at the center especially of the human security agenda for some time, governments and societies still seem to be unprepared for the challenges that lie ahead. We would argue that this is also a result of the dominant narrative of health security and its lack of basing its dominant logic of action on a normative framework that opens up a long-term systemic perspective without losing sight of individuals as bearers of duties and rights. Therefore, we suggest an alternative narrative of global health that does not rest on a logic of exceptionalism but on the logic of solidarity (see Fig. 2).

Solidarity as an ethical principle has been discussed for some time in global health (e.g. Harmon 2006) and is explicitly discussed with view to the COVID-19 pandemic (de Campos 2020). In fact, Frenk, Gómez-Dantés and Moon argued, that the existing concept of global health is not able “to capture the essence of globalization” (Frenk et al. 2014, p. 94), and suggested to reframe it as “health of the global population” and “product of health interdependence” (Frenk et al. 2014, p. 95), thereby using the term solidarity “to refer to situations of interdependence created by the complex division of roles characteristic of modern societies” (Frenk et al. 2014, p. 97). In political terms, solidarity relates to “individuals performing reciprocal duties and respecting reciprocal rights” (Harmon 2006, p. 217). From this definition, Harmon derives several propositions of what solidarity comprises: The recognition that individuals are embedded in social contexts, the focus on the well-being of others thus emphasizing equality and the promotion of welfare, and the demand for common action (Harmon 2006, p. 218).

Another defining feature of solidarity is that the distinction between “we” and “them” is suspended, since solidarity is exercised within a symmetrical relationship characterized by equality. Usually this works quite well within groups. But what about solidarity between groups, especially those which are geographically more distant? With respect to global health, the common experience of vulnerability may act as source of solidarity (West-Oram and Buyx 2017, p. 215–216). From this perspective, solidarity can be understood “as enacted practices that are based on concrete recognition of similarity in a given *specific* context” (West-Oram and Buyx 2017, p. 213; emphasis in the original).

Vulnerability is a crucial factor for how individuals and societies cope with risks. The current COVID-19 pandemic, however, teaches us that societies across the globe are facing shared vulnerabilities (Gostin 2017) since our economies and many aspects of our lifestyles have become so interdependent as the example of global tourism shows. Historically, it used to be the case that the wealthier people in the Global North had the privilege of ever-growing safety from infectious diseases with which people in the Global South still had to cope. However, even if countries of the Global North may still have more resources available to cope with newly emerging health threats like COVID-19, the degree of vulnerability they are encountering has considerably increased (West-Oram and Buyx 2017, p. 206). Therefore, the alternative narrative of global health we propose does not start from health threats and emergencies but sketches a different setting of shared vulnerabilities and interdependence from which to proceed.

Taking vulnerabilities as the starting point, will also lead to a different characterization of the main protagonists. The COVID-19 pandemic permeates all borders and affects all countries. Yet, it has unequal effects on livelihoods across all societies. Overcoming dichotomies of “we” and “them” and thinking instead in terms of vulnerabilities pays tribute to the inequalities people around the globe suffer. Focusing on inequality and justice puts individuals at the center who have a right to health and who also have the agency to realize it as duty bearers and rights holders. Although states, which ultimately are the stewards of securing human rights for their citizens, are not the main protagonists anymore, they still have a crucial role to play, but in conjunction with IOs, private and civil-society actors. They all share the responsibility—albeit in a differentiated way—to provide health as a global public good.

Consequently, from a perspective of countries of the Global North, infectious diseases should not be looked upon as diseases originating in countries of the Global South that have to be contained. Instead, the emplotment of the narrative leads to a different causal origin of the problem societies and individuals are facing: The social determinants of health have long been acknowledged (Commission on Social Determinants of Health 2008) and the imperative of “health equity” that it implies. Thus, health inequities are seen as causes of vulnerabilities.

At the same time, the awareness of the global nature of the pandemic leads to calls for collective and multilateral action on the one hand and to overcoming traditional perceptions and categories of countries-in-need on the other. Ultimately, health as a global public good can only be attained by “building more robust global institutions for pooling risks, resources, and responsibilities among sovereign states—and in many cases, also non-state actors” (Frenk et al. 2014, p. 96). The current push for a vaccine against the SARS-CoV‑2 virus will lead to quite different results if framed in terms of national security or in terms of global solidarity, which the WHO and the United Nations have been advocating constantly. Humankind will only overcome the current and future pandemics if health is not regarded as an instrument for achieving security or economic development, but as a human right, a value in its own right and goal of policies.

## Conclusion

We began this contribution with the premise that narratives shape how people perceive the world around them and condition how political actors are framing policy choices to cope with challenges. From the onset, the current Coronavirus pandemic has been embedded in a narrative of global health security. Consequently, it has been framed as an exceptional threat that had to be countered with a range of far-reaching emergency measures.

**Fig. 3:**
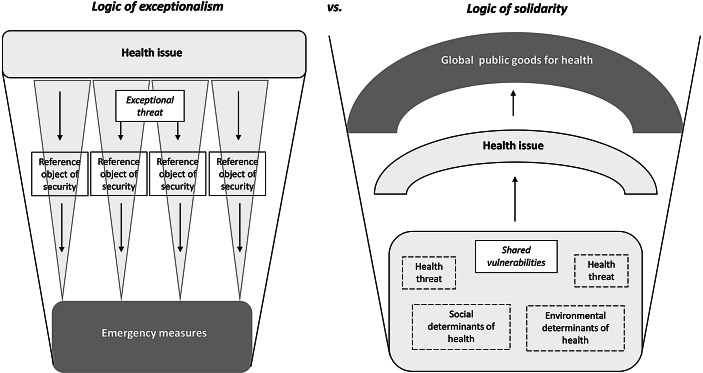
Differing narratives of global health: Logics of action and their consequences. (Source: Authors)

Although the narrative of global health security is characterized by two distinct variants, with the concept of human security opening up the space to think of how individuals and their rights are affected by inequalities, both variants are firmly rooted in depicting health threats as exceptional and existential to our lives. COVID-19 is a prime example of how the logic of exceptionalism is shaping and limiting the responses to it: The range of policy choices is still primarily focused on emergency measures (see Fig. 3).

The current pandemic, however, provides the opportunity to think in terms of shared vulnerabilities as context for tackling the disease. These vulnerabilities are not only due to changing global health threats. They are also caused by social and environmental determinants of health. The more the consequences of climate change, for example, are felt all over the world, the more, even countries of the Global North, realize how hard it will be for them to adapt to it. We have argued that the awareness of shared vulnerabilities allows for a logic of solidarity to be set in motion.

Reframing the narrative of global health from security to solidarity with the aim of providing global public goods for health will change the “rules of the game”: Only then the intersectional and interdependent nature of health as a product of its social determinants and ecological environment will translate into political action guided by a long-term systems approach based on prevention, surveillance and health systems strengthening to enhance the resilience of communities.
